# Supplementary material for: Cord-Blood-Stem-Cell-Derived Conventional Dendritic Cells Specifically Originate from CD115-Expressing Precursors
Source: Cancers (Basel). 2019 Feb 5;11(2):181. doi: 10.3390/cancers11020181 (PMC6406310; doi:10.3390/cancers11020181)
Supplement: Supplementary file 1 [file cancers-11-00181-s001.zip › cancers-414525-suppl/cancers-414525-supplementary-Figures.docx]

**Supplementary Materials: Cord-Blood-Stem-Cell-Derived Conventional Dendritic Cells Specifically Originate from CD115-Expressing Precursors**

**Maud Plantinga^1^, Colin G. de Haar^2^, Ester Dünnebach^1^, Denise A.M.H. van den Beemt^1^, Kitty W.M. Bloemenkamp^3^, Michal Mokry^4^, Jaap Jan Boelens^1,5,6^ and Stefan Nierkens^1,5^***


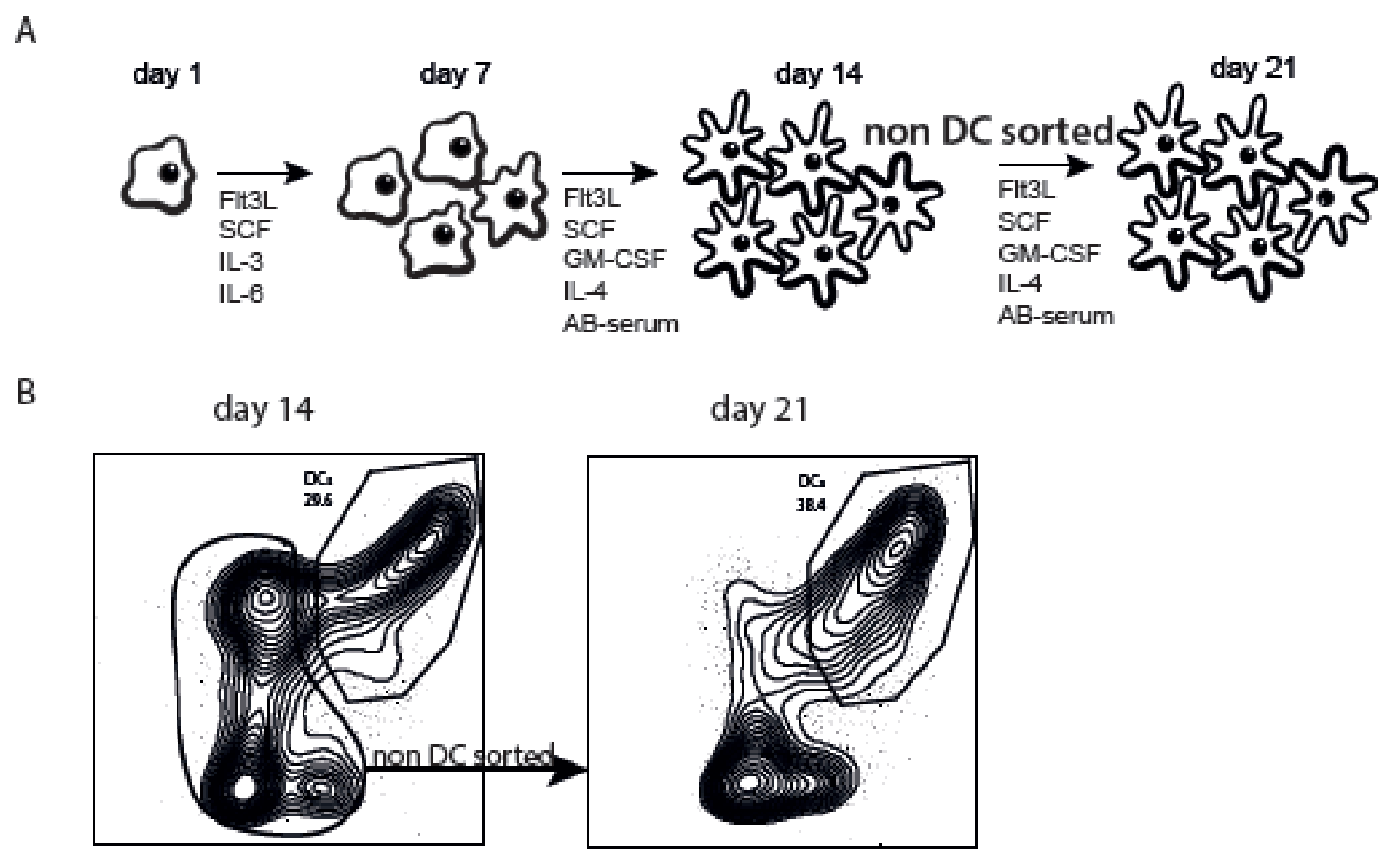


**Figure S1.** Differentiation of the non-DC fraction. (**A**) The culture protocol for the generation of CB-DCs and the second differentiation after sorting the non-DCs at day 14. (**B**) CD11c and HLA-DR expression on alive cells after an additional differentiation.


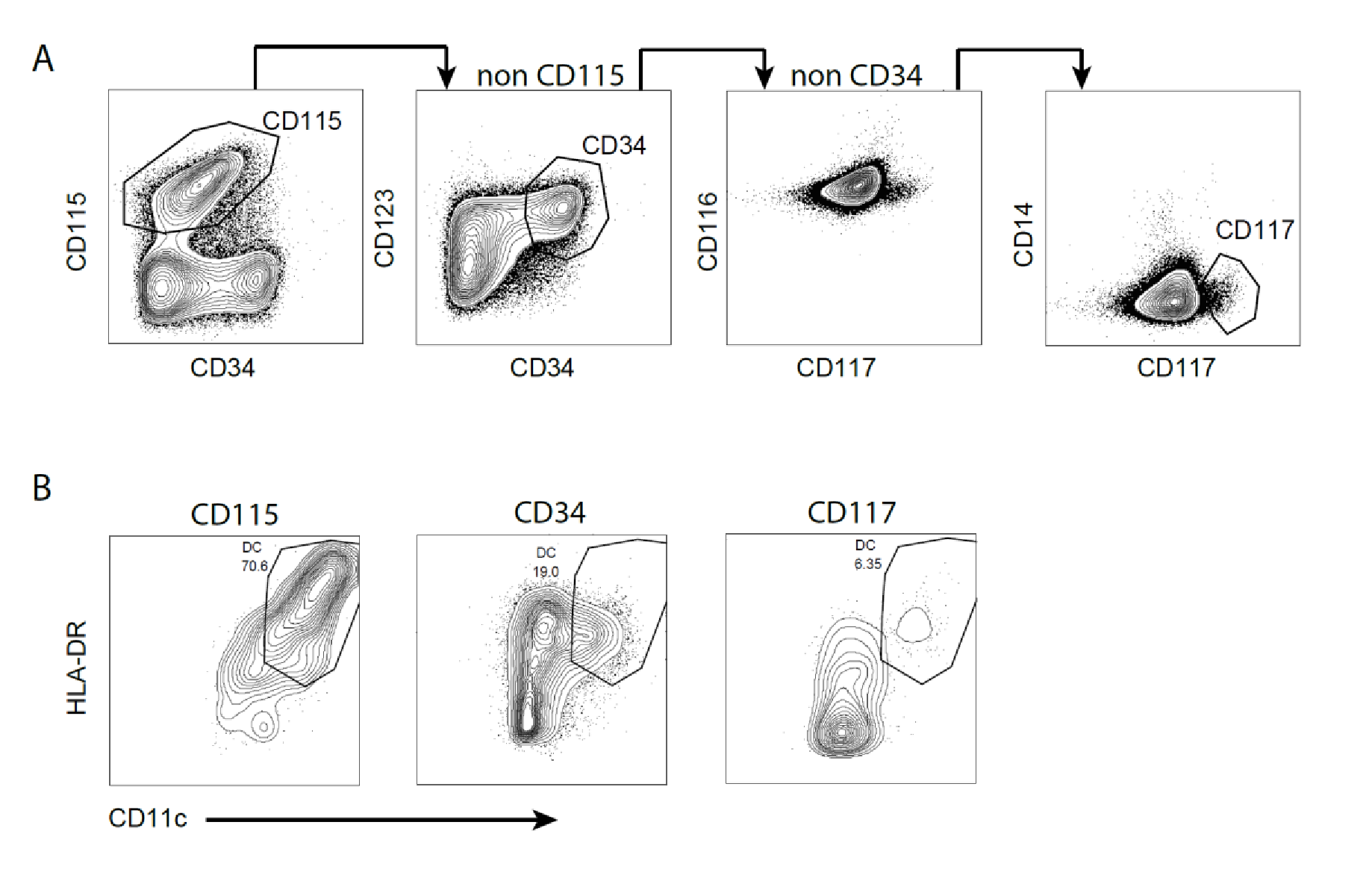


**Figure S2.** Different progenitors found in CB culture. (**A**) Gating strategy to identify myeloid progenitors within the CB culture at day 7 based on their expression of cytokine and growth factor receptors. (**B**) The expression of CD11c and HLA-DR at day 14 after differentiating the sorted progenitors in differentiation medium. Data represent at least two independent experiments.


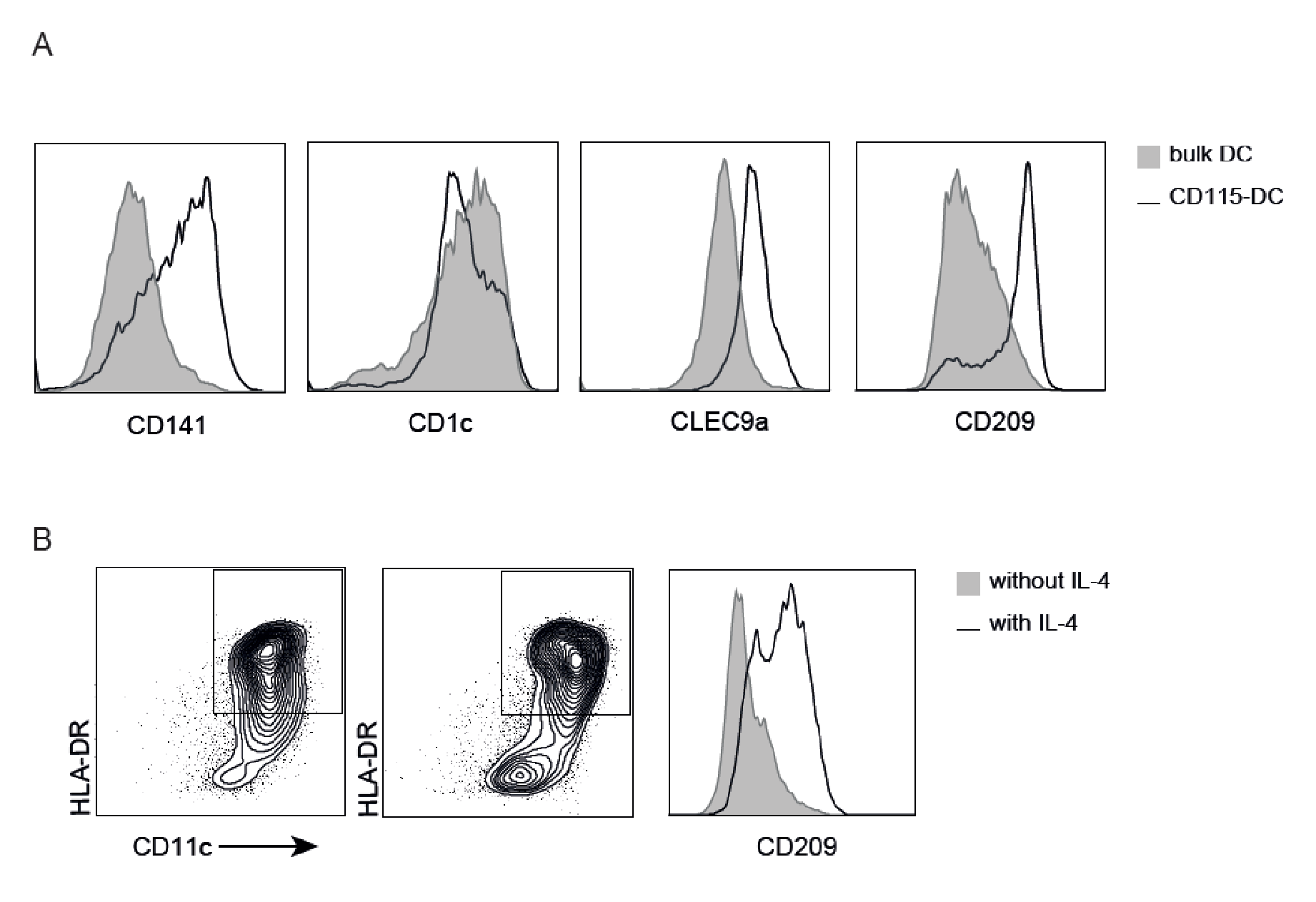


**Figure S3.** CD209 and CLEC9A expression after differentiation. (**A**) The expression of CD141, CD1c, CLEC9A, and CD209 gated within CD11c+HLA-DR+/live/single cells after differentiating either bulk or sorted CD115^+^ precursors for 14 additional days with the protocol described by Poulin et al. (**B**) The expression of CD11c and HLA-DR at day 14 after differentiating the sorted progenitors in differentiation medium with (left) or without IL-4 (middle) and the loss of CD209 in the absence of IL-4 (right). Data represent at least two independent experiments.


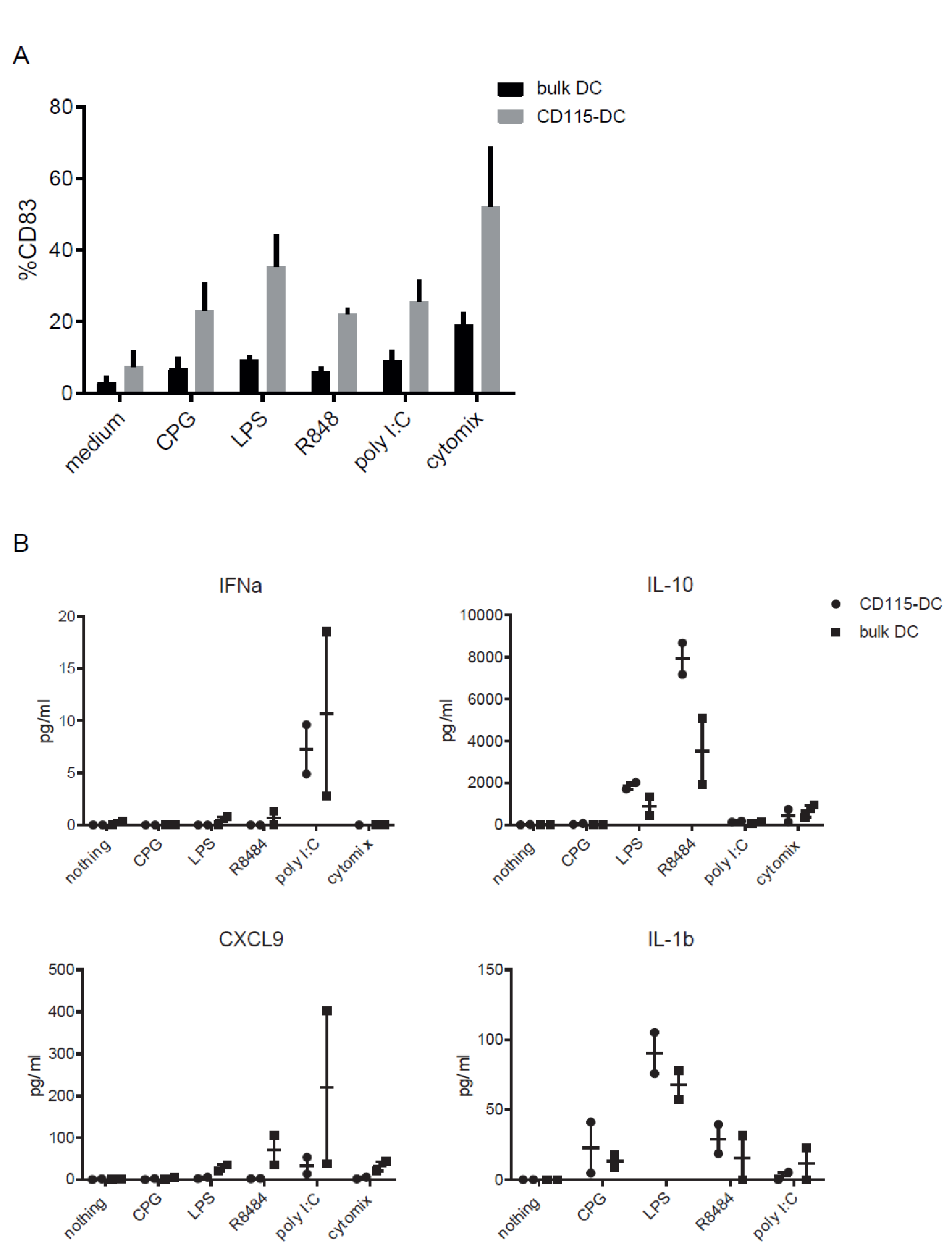


**Figure S4.** DCs stimulated with different TLR ligands. (**A**) The percentage CD83 gated as CD83+/CD11c+HLA-DR+/live/single cells after o/n maturation with indicated TLR ligands or medium as a control comparing bulk DCs to CD115-DCs. (**B**) IFNa, IL-10, CXCL9, and IL-1b production, measured in the supernatant using luminex from bulk DC and CD115-DC cultures.

#
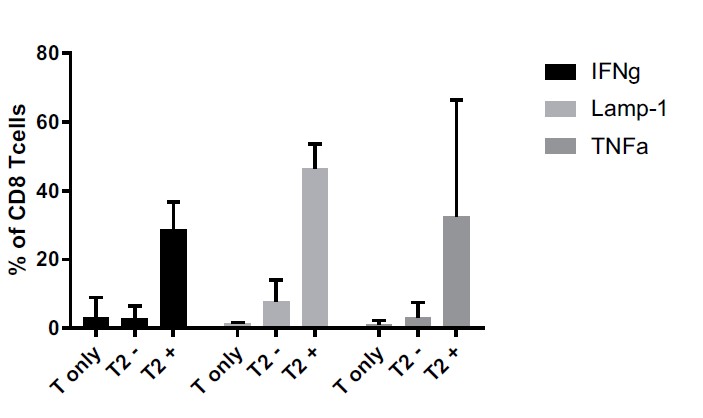


**Figure S5.** WT1-specific T-cell assay controls. IFNγ and TNFα production and Lamp-1 expression by WT1-specific T cells unstimulated (T only) or stimulated with unloaded (-) or WT1-loaded (+) T2 30 cells. Controls for the WT1-specific T-cell assay in Figure 4.
